# Involvement of nucleic acid-sensing toll-like receptors in human diseases and their controlling mechanisms

**DOI:** 10.1186/s12929-025-01151-9

**Published:** 2025-06-10

**Authors:** You-Sheng Lin, Yung-Chi Chang, Tzu-Yu Pu, Tsung-Hsien Chuang, Li-Chung Hsu

**Affiliations:** 1 Institute of Molecular Medicine, National Taiwan University, No 7, Chung Shan S. Rd., Taipei, 10002 Taiwan; 2 Cardiovascular and Mitochondrial Related Disease Research Center, Hualien Tzu Chi Hospital, Buddhist Tzu Chi Medical Foundation, Hualien, Taiwan; 3 Graduate Institute of Immunology, National Taiwan University, Taipei, Taiwan; 4 Center of Precision Medicine, College of Medicine, National Taiwan University, Taipei, 100 Taiwan

**Keywords:** Toll-like receptors, Nucleic acid-sensing, Human disease

## Abstract

The innate immune system is the host’s initial response to eliminate pathogens and repair tissue damage. Innate immune cells, such as macrophages and dendritic cells, use pattern recognition receptors (PRRs) to recognize microbial structures and stress-induced molecules released from dead or damaged cells, thereby initiating immune responses. Among PRRs, Toll-like receptors (TLRs) are well-studied and are located either on the cell surface or in endosomal compartments. Most endosomal TLRs specifically recognize nucleic acids and are thus referred to as nucleic acid (NA)-sensing TLRs. Upon activation, these receptors induce the production of inflammatory cytokines and type I interferons and initiate subsequent adaptive immunity. These immune responses work to suppress pathogens and inhibit tumor growth. However, excessive cytokine and interferon production can lead to various inflammatory diseases. This review focuses on mammalian nucleic acid-sensing TLRs, summarizing the molecular regulation of their activations, the impact of their dysregulation on human diseases, and therapeutic strategies that target these TLRs.

## Introduction

**Fig. 1:**
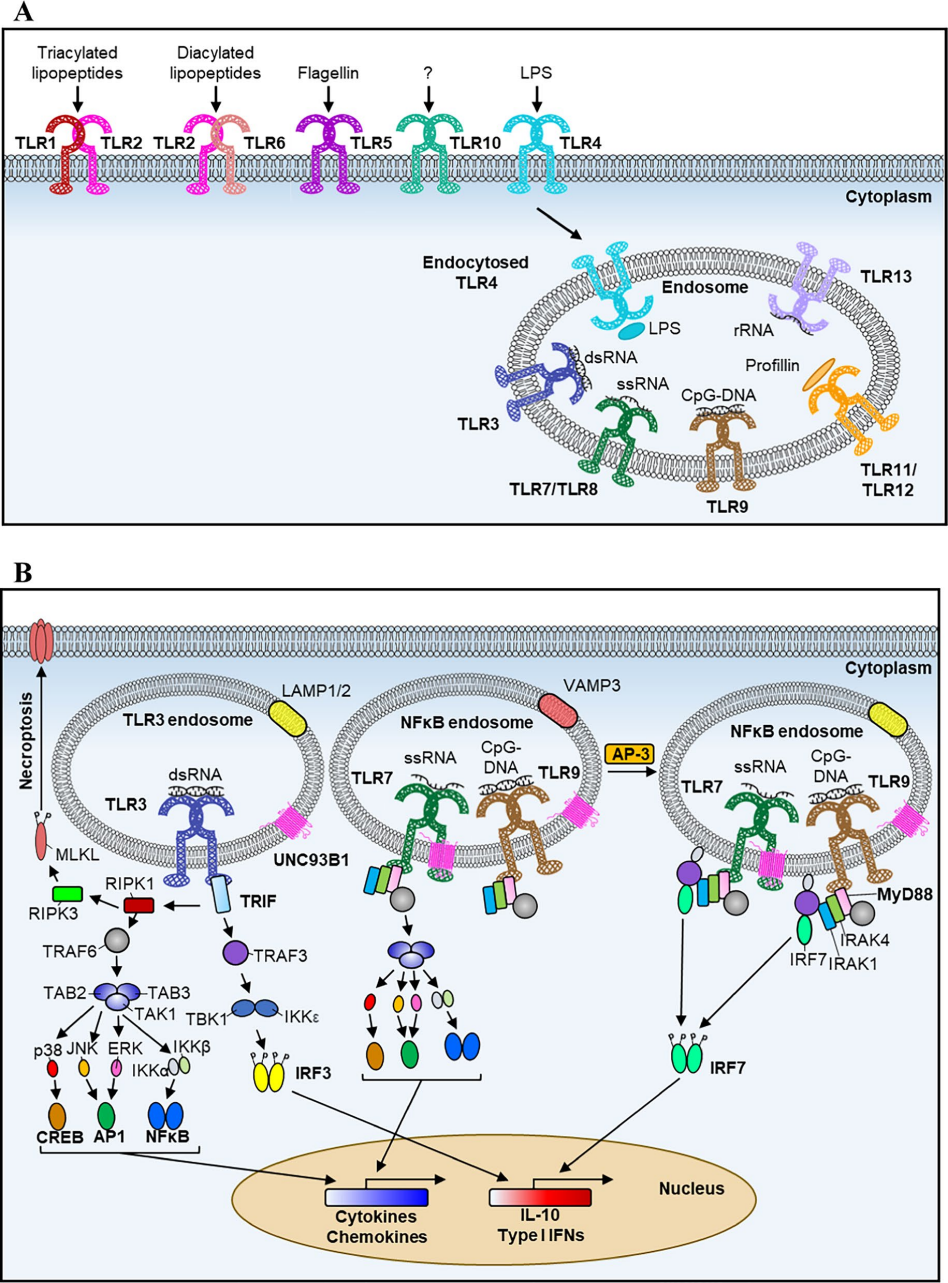
The cellular localization of mammalian TLRs and their PAMPs and NA-sensing TLR signaling pathways. **A** Mammalian cells have 13 identified TLRs. TLR1, TLR2, TLR4, TLR5, TLR6, and TLR10 localize at the plasma membrane, where they recognize PAMPs from extracellular spaces. Conversely, TLR3, TLR7, TLR8, TLR9, and TLR11-13 reside in endosomal compartments, where they encounter their ligands internalized from outside the cells. **B** Upon sensing dsRNA, TLR3 recruits the adaptor protein TRIF, which induces the activation of NF-κB and IRF3, leading to the production of proinflammatory cytokines, chemokines, type I IFNs, and IL-10. NF-κB activation is mediated by RIPK1 and TRAF6, which subsequently recruit the TAK1/TAB2/TAB3 complex, activating TAK1. TAK1 then activates the IKK complex and MAPKs, leading to the activation of transcription factors NF-κB, AP-1, and CREB. Additionally, the TLR3-TRIF complex activates TRAF3, which associates with and activates TBK1 and IKKε. TBK1 and IKKε subsequently phosphorylate and activate IRF3. Moreover, TLR3-TRIF triggers necroptosis through RIPK3, which phosphorylates MLKL. TLR7 and TLR9 are mainly expressed in pDCs. Upon activation by their ligands, TLR7 and TLR9 activate NF-κB in early endosomes through the formation of a signalosome containing MyD88, IRAK1, and IRAK4. This complex subsequently associates with TRAF6, resulting in NF-κB activation. With the assistance of the AP-3 complex, the TLR7/9-MyD88-IRAK signalosome is transported to mature lysosome-related organelles (LROs), where it recruits TRAF3, inducing the production of type I IFNs

The innate immune system is the initial response in the host to eliminate invading pathogens and repair subsequent tissue damage. During microbial infections, innate immune cells, including macrophages and dendritic cells (DCs), employ germline-encoded pattern recognition receptors (PRRs), including Toll-like receptors (TLRs), to recognize microbial structures, such as carbohydrates, lipids, proteins, and nucleic acids, together named PAMPs (pathogen-associated molecular patterns), or endogenous molecules including proteins, DNAs, and RNAs, called damage-associated molecular patterns (DAMPs), released from stressed or damaged tissues and then to mount a defensive response [31, 150]. TLRs are the most characterized molecules among the PRRs and play an important role in the innate immune system [86, 150]. TLRs were identified in vertebrates based on a conserved domain termed Toll/IL-1 receptor (TIR) domain that is shared with Toll proteins in *Drosophila melanogaster* [178]. Tolls and TLRs proteins are type I transmembrane proteins composed of N-terminal leucine-rich repeats (LRRs) and a transmembrane region followed by a cytoplasmic TIR domain at C-terminus. To date, thirteen TLRs [10 in humans (TLR1–TLR10) and twelve in mice (TLR1–TLR9 and TLR11–TLR13)] have been identified in mammals (Fig. 1A). Based on their cellular localization, TLRs can be categorized into two subgroups: one group of TLRs, including TLR1, TLR2, TLR4, TLR5, TLR6, and TLR10, is localized at the cell surface; the other group, composed of TLR3, TLR7, TLR8, TLR9, and TLR11-13, is expressed in intracellular organelles, including endosomes and endolysosomes [86, 149, 151]. Most of their ligands, both PAMPs and DAMPs, have been characterized, and interestingly, most endosomally localized TLRs, including TLR3, TLR7, TLR8, TLR9, and TLR13, specifically recognize nucleic acids. Therefore, they are also called nucleic acid (NA)-sensing TLRs [31, 149]. Two main signaling pathways are induced after TLR activation: nuclear factor kappa-B (NF-κB) and interferon regulatory factor 3 (IRF3) or IRF7 signal pathways for the production of inflammatory cytokines and type I interferons (IFNs), respectively. Cytokines and IFNs convey signals to nearby cells, triggering the expression of an array of antiviral and antibacterial genes, thereby suppressing pathogen replication and dissemination. However, excess production of cytokines, IFNs, and other mediators has been associated with inflammatory diseases, autoimmune diseases, and cancer [149, 205, 222]. Various mechanisms have been identified that tightly regulate TLR signaling pathways, preventing uncontrolled inflammation. In this review, we will mainly focus on mammalian NA-sensing TLRs—TLR3, TLR7, TLR8, and TLR9—to summarize current knowledge on the regulatory mechanisms governing their activation, examine the implications of their dysregulation in human diseases, and discuss therapeutic applications targeting these TLRs.

## Ligand recognition and control mechanisms of NA-sensing TLR activations

Mammalian NA-sensing TLRs recognize ligands with nucleic acid-related structures: TLR3 detects double-stranded RNA (dsRNA), TLR7 and TLR8 recognize single-stranded RNA (ssRNA), and TLR9 identifies DNA with unmethylated CpG motifs [7, 120, 122, 227]. These TLRs are synthesized in the endoplasmic reticulum (ER) and then transported to endosomal compartments, where they encounter and are activated by their respective agonists, typically derived from pathogens or dead cells [86, 149]. These TLRs are essential for host defense against infections. Nevertheless, dysregulation of TLR signaling has been linked to various inflammatory and autoimmune diseases [197]. Consequently, multiple mechanisms exist to regulate these nucleic acid-sensing TLRs for their precise activation. These include ligand recognition to initiate signal transduction, regulation of endosomal trafficking, proteolytic processing of the receptors, and negative regulation of the signaling pathways.

## Ligand recognition of NA-sensing TLRs

TLR3 was first identified as a receptor for synthetic double-stranded (ds)RNA, such as polyinosinic:polycytidylic acid (poly(I:C)), which activated TLR3-mediated NF-κB activation in cell-based assays and induces cytokine production, including TNFα, IL-6, and IL-12 in splenocytes from wild-type mice. In contrast, the immunostimulatory effect of poly(I:C) on cytokine production was absent in cells from TLR3 knockout mice [7]. Notably, for viral detection, TLR3 prefers dsRNA strands longer than 40 bp, a feature rarely found in mammalian cells [180]. Human TLR7 and TLR8 recognize synthetic chemical compounds, such as imidazoquinolines and nucleoside analogs, as well as viral ssRNA [120, 121, 335]. These two TLRs are the most closely related in the phylogeny of all TLRs, leading to overlapping ligand and viral recognition [47]. For example, imidazoquinoline compounds like R848 and CL097 are able to activate both TLR7 and TLR8, while imiquimod and gardiquimod preferentially activate TLR7. Similarly, guanosine-uridine (GU)-rich ssRNAs activate both TLR7 and TLR8, whereas adenosine-uridine (AU)-rich ssRNA preferentially activate TLR8 [63, 102, 202]. Although mouse TLR7 shares a similar ligand recognition profile with human TLR7, rodent TLR8s (including mouse, rat, and rabbit) show a reduced response to ligands compared to their human homolog, likely due to variations in the ectodomain of these TLR8s [187]. Recent research on the structures of TLR7 and TLR8 has uncovered the existence of two distinct binding sites for their RNA ligands: site 1 is highly conserved and recognizes specific nucleotides (guanosine for TLR7 and uridine for TLR8), while site 2 is not conserved and binds to a short RNA fragment [290, 291, 336]. Both the binding of ssRNA and nucleotides are crucial for optimal activation of TLR7 and TLR8 activation. TLR9 is activated by synthetic CpG-containing oligodeoxynucleotides (CpG-ODNs) and hypomethylated CpG-DNA derived from viruses [21, 122]. Hypomethylated CpG DNA motifs are not commonly present in the mammalian genome. This phenomenon of ligand recognition is an example of how these endosomal TLRs selectively recognize nucleic acids derived from microorganisms. Similar to TLR7 and TLR8, TLR9 possesses two binding sites: one that interacts with CpG DNA and another that binds cytosine-containing DNA fragments, both working cooperatively to promote dimerization and activation of TLR9 [226]. Furthermore, lysosomal endonuclease RNase T2, along with 5ʹexonuclease phospholipase (PLD) and DNase II, plays a crucial role in generating specific ligands that preferentially activate TLR7 and TLR9, respectively [24, 40]. 

## NA-sensing TLR signaling

Upon engagement, TLRs rapidly activate both the mitogen-activated protein kinases (MAPKs)/IKK/NF-κB and the IRF3/7 signaling pathways, leading to the production of proinflammatory cytokines and type I IFNs [86, 149]. Adaptor proteins, myeloid differentiation factor 88 (MyD88) or TIR domain-containing adaptor-inducing IFNβ (TRIF), associate with TLRs when they engage NA ligands [31, 86]. TLR3, found in both immune and nonimmune cells, is the sole TLR signaling exclusively through the adaptor protein TRIF [31]. Unlike TLR4, the TRIF signaling pathway activated by TLR3 does not require the TRIF-related adaptor molecule (TRAM). TLR3 specifically recognizes viral dsRNA and binds to TRIF, which then recruits receptor-interacting protein kinase 1 (RIPK1) through the RIP homotypic interaction motif (RHIM) at its C-terminus [143]. RIPK1 then associates with tumor necrosis factor receptor-associated factor 6 (TRAF6). Additionally, TRIF recruits TRAF3 to the TLR3 complex. Once activated, TRAF3 and TRAF6 induce the activation of TANK-binding kinase 1 (TBK1), IKK, and MAPKs, which in turn activate the transcription factors IRF3, NF-κΒ, and activating protein 1 (AP-1), thereby initiating a rapid antiviral response to defend against various types of viral infection [31, 298, 305, 314]. In addition, TRIF can trigger necroptosis by recruiting receptor-interacting protein kinase 3 (RIPK3) via its RHIM domain, which subsequently phosphorylates mixed lineage kinase domain-like protein (MLKL) within TLR3 signaling [144]. The phosphorylated MLKL then translocates to the plasma membrane, where it oligomerizes, resulting in a loss of membrane integrity. TLR7, TLR8, and TLR9 (collectively known as TLR7/8/9) form a subfamily of TLRs due to their close relation compared to other TLRs [151]. TLR7 and TLR9 are primarily expressed in pDCs, while TLR8 is predominantly found in myeloid cells, including monocytes, macrophages, and myeloid DCs (mDCs) [142, 151]. pDCs are capable of producing large amounts of type I IFNs in response to viral infections [151, 186]. In these cells, the engagement of TLR7, TLR8, and TLR9 triggers the formation of a MyD88-containing complex, known as the Myddosome (Fig. 1B). This complex, composed of MyD88, interleukin-1 receptor-associated kinase 4 (IRAK4), and TRAF6, activates the IRF7 and NF-κB signaling pathways [103, 173, 315]. In the IRF7 signaling pathway, TRAF3, IRAK1, IKKα, osteopontin (OPN), and phosphoinositide 3-kinase (PI3K) are reported to be necessary for IRF7 activation, although the detailed underlying mechanism remains unclear [107, 129, 270, 300]. In the NF-κB signaling pathway, TRAF6 is recruited to the MyD88-IRAK4 complex and becomes activated. Activated TRAF6 subsequently ubiquitylates the protein kinase transforming growth factor-β-activated kinase 1 (TAK1), leading to the activation of IKK/NF-κB and MAPKs (Fig. 1B).

## Regulation of NA-sensing TLR trafficking and proteolytic processing

NA-sensing TLRs encounter their ligands derived from microbes after pathogens are internalized and degraded within endosomal compartments [19]. This mechanism prevents these receptors from recognizing self-derived ligands in the endoplasmic reticulum (ER) and the Golgi apparatus [20]. All NA-sensing TLRs are translated and glycosylated in the ER and Golgi, and they are subsequently transported to the endosomal compartments with the assistance of unc-93 homolog B1 (UNC93B1), a 12 trans-membrane chaperone protein [157, 172, 284]. Interestingly, different NA-sensing TLRs likely employ distinct trafficking routes to reach endosomes, although these processes are all mediated by UNC93B1 [171, 172]. For example, TLR7 directly traffics from the Golgi to the endosomes through the adaptor protein complex 4 (AP-4), whereas TLR9 is first transported to the plasma membrane, where it undergoes endocytosis into the endosomes facilitated by AP-2 [172]. TLR3, unlike other NA-sensing TLRs, relies on intracellular S100A9, a calcium-binding protein, to facilitate its localization to late endosomes for ligand detection and activation [298]. Recent studies revealed that UNC93B1 maintains its association with TLR7 after TLR7 ligation in the endosomes, subsequently becoming ubiquitinated. This ubiquitination recruits syntenin-1, which sorts receptor cargos into intraluminal vesicles (ILVs), resulting in the termination of receptor signaling [198]. Conversely, UNC93B1 must dissociate from TLR3 and TLR9 in endosomes to allow ligand binding and initiate downstream signaling [199]. Cryo-electron microscopy (Cryo-EM) structural investigations confirmed that TLR3 is unable to dimerize while bound to UNC93B1 due to steric interference between the two UNC93B1 molecules, whereas UNC93B1 and TLR7 form a 2:2 complex [137]. Moreover, two recent reports indicate that several human genetic variants of UNC93B1 may contribute to the development of autoimmune diseases, likely due to enhanced activation of TLR7 and, to a lesser extent, TLR8 [6, 59].

Accumulated evidence suggests that the cellular localization of NA-sensing TLRs is crucial in determining cytokine production. In pDCs, TLR7 and TLR9 initiate distinct signaling pathways from two separate endosomal compartments, termed NF-κB endosomes and IRF7 endosomes to produce proinflammatory cytokines and type I IFNs, respectively [25, 258]. The adaptor complex AP-3, which is involved in protein trafficking, interacts with TLR9 and possibly TLR7, facilitating their movement from NF-κB endosomes to specialized lysosome-related organelles (LROs) for IRF7 activation and the production of type I IFNs. However, more studies indicate that different mechanisms mediate the trafficking of TLR7 and TLR9 to LROs. For example, the GTPase ARL8B is required for TLR7-driven type I IFN production but is dispensable for TLR9-induced type I IFN production [253]. In addition, phosphatidylinositol 3-phosphate 5-kinase is necessary for TLR9 trafficking but not for TLR7 to LSOs [117]. In contrast to TLR7 and TLR9, TLR3 requires the endolysosomal compartment, formed by the fusion of late endosomes with lysosomes to drive cytokines and type I IFN production [298]. Notably, while AP-3 is partially involved in TLR3-triggered type I IFN expression, its involvement in TLR3 trafficking to the endolysosomal compartment remains unclear [258]. Furthermore, the mammalian target of rapamycin complex 1 (mTORC1) is required for NA-sensing TLR-mediated type I IFN production [34, 263]. Interestingly, another investigation revealed that ligand-activated TLR3 recruits mTORC2, facilitating the transport of TLR3-containing vesicles to the cell periphery. At this location, TLR3 interacts with TRAF3 and mTORC1 to induce the production of type I IFNs [259]. Nevertheless, the precise mechanisms regulating the trafficking and localization of NA-sensing TLRs, as well as their downstream signaling pathways, are not yet well understood.

NA-sensing TLRs need to undergo proteolytic processes of their extracellular LRR domains within endosomal compartments to recruit the adaptor proteins MyD88 or TRIF and initiate downstream signaling upon ligand engagement [78, 79, 94]. This proteolytic cleavage serves as a critical regulatory checkpoint, ensuring that only cleaved TLRs become activated upon encountering ligands obtained from endocytosis or phagocytosis, thereby preventing self-recognition within the ER and Golgi apparatus. Numerous studies have highlighted the significant role of lysosomal cathepsins and asparagine endopeptidases (AEP) in the cleavage of NA-sensing TLRs, with the exception of human TLR7 and TLR8 [78, 94, 125, 138, 241, 266]. Most NA-sensing TLRs undergo multistep proteolytic cleavages: first, a large portion of the ectodomain is removed by AEP and cathepsins, followed by further trimming by cathepsins. However, the cleavage of human TLR7 and TLR8 is mediated by furin-like proprotein convertase at neutral pH before their delivery to acidic endosomes, highlighting a distinct regulatory mechanism for these receptors.

## Negative regulation of NA-sensing TLR signaling

In addition to receptor processing, localization, and ligand availability, several mechanisms regulate NA-sensing TLR signaling pathways. One such mechanism involves the production of soluble TLR ectodomains, which contribute to the termination of TLR signaling. For example, the human soluble TLR9 ectodomain is generated through proteolytic cleavage, resulting in a form that lacks the TIR domain. This soluble TLR9 acts as a competitive inhibitor by binding to its ligand or the full-length receptor, preventing receptor dimerization and thereby downregulating downstream signaling [44]. Besides soluble TLR ectodomains, molecules induced upon TLR activation, such as the deubiquitinating enzyme A20 and suppressor of cytokine signaling 1 (SOCS1), also play critical roles in providing negative feedback regulation [217, 299]. A20 removes K63-linked polyubiquitin chains from TRAF6, effectively terminating TLR3- and TLR9-driven NF-κB activation, while SOCS1 inhibits IRAK-1 phosphorylation following TLR9 engagement. Ubiquitination has been shown to play a crucial negative regulator of NA-sensing TLR signaling. For example, the E3 ubiquitin ligase TRIAD3A (RNF216) catalyzes K48-linked polyubiquitination on TLR9, targeting it for proteasomal degradation and thereby reducing receptor levels [48]. Tripartite Motif Containing 28 (TRIM28), an E3 ubiquitin ligase, catalyzes the addition of K48-linked polyubiquitin chains to TRIF, marking it for proteasomal degradation and attenuating TLR3 signaling, leading to a reduction in type I IFN expression in response to TLR3 activation [132]. Similarly, TRIM21, an autoantigen associated with Systemic Lupus Erythematosus (SLE), negatively regulates type I IFN production driven by TLR3, TLR7, and TLR9 [123, 124]. Pellino-3, another E3 ubiquitin ligase, has been reported to inhibit TLR3 signaling by promoting the ubiquitination of TRAF6, which in turn blocks TRAF6-facilitated phosphorylation and activation of IRF7, thereby reducing the induction of type I IFNs [272]. Our recent study identified an E3 ubiquitin ligase, Zinc and Ring Finger 1 (ZNRF1), which catalyzes K63-linked polyubiquitination of TLR3. This modification promotes TLR3 lysosomal trafficking and degradation, prolonging TLR3 signaling and enhancing type I IFN production [185]. Mice deficient in ZNRF1 show resistance to encephalomyocarditis virus (EMCV) infection due to increased type I IFN production, but they suffer exacerbated lung barrier damage triggered by antiviral immunity, leading to enhanced susceptibility to secondary respiratory bacterial infections. This highlights the importance of proper TLR3 signaling termination in preventing secondary bacterial superinfection. Interestingly, ZNRF1 does not directly ubiquitinate TLR7 or TLR9 (unpublished data). However, a recent report showed that UNC93B1 undergoes K63-linked polyubiquitination at lysine 333, facilitating the recruitment of syntenin-1, which promotes TLR7 sorting into ILVs of multivesicular bodies, thereby terminating TLR7 signaling [198]. The E3 ubiquitin ligase responsible for this modification of UNC93B1 remains unidentified, as does the mechanism by which TLR9 signaling is terminated.

## NA-sensing TLR activations and human disease

Activation of NA-sensing TLRs is known to initiate antiviral and antitumor immune responses. These receptors recognize viral and tumor-associated molecular patterns, triggering signaling pathways that lead to the production of cytokines and other immune mediators essential for combating infections and tumors. This immune response plays a crucial role in clearing harmful agents and promoting tissue repair, making it a key defense mechanism for maintaining health [81, 304]. Acute inflammation typically resolves quickly after pathogen elimination and tissue recovery [42, 321]. However, chronic inflammation is a well-known factor in tumor promotion. Additionally, persistent inflammation can disrupt immune tolerance, cause tissue damage, and contribute to the onset of autoimmune diseases, such as psoriasis, rheumatoid arthritis, and SLE [211, 297]. The roles and mechanisms of NA-sensing TLR activation in host defense, therapeutic effects, and disease pathogenesis are complex and multifaceted.

## Roles of NA-sensing TLRs in host defense against viral infections

TLR3 senses viral dsRNA to initial host immune responses against viral infections. Viral genomic dsRNA from type I Lang mammalian reovirus was shown to upregulate cluster of differentiation 69 (CD69) expression on wild-type B cells, while TLR3-deficient cells exhibited no response to the viral dsRNA [7, 60, 262]. Additionally, TLR3 has been reported as the receptor for intermediates generated during the life cycle of single-stranded (ss) RNA viruses, which include rhinovirus (RV), West Nile virus (WNV), Japanese encephalitis virus (JEV), dengue fever virus (DENV), chikungunya virus (CHIKV), respiratory syncytial virus (RSV), Influenza A virus (IAV), enterovirus (EV), hepatitis C virus (HCV), Coxsackie virus group B3 (CVB3) [161, 183, 235, 305, 333]. Notably, children with inherited TLR3 deficiency are more susceptible to these viral infections. For examples, severe influenza pneumonitis has been associated with autosomal dominant (AD) TLR3 deficiency, due to impaired TLR3-dependent production of IFNs [183]. Furthermore, AD TLR3 deficiency has also been linked to increased susceptibility to Enterovirus A71 (EV71), which causes a broad spectrum of childhood diseases [161].

In addition, dsRNA intermediates can be produced by viruses with dsDNA genomes. TLR3 has been reported to respond to dsDNA viruses such as herpes simplex virus (HSV), Epstein-Barr virus (EBV), Kaposi’s Sarcoma-associated herpesvirus (KSHV), and Vaccinia virus (VACV). EBV and KSHV are transmitted through close contact and can lead to lymphoproliferative diseases or lymphomas [35, 134, 257, 259]. In humans, single nucleotide polymorphisms (SNPs) in the TLR3 gene have been associated with susceptibility to infections of HSV-1, CVXB3, human T-lymphotropic virus type 1 (HTLV-1), HCV, HBV, Zika, and SARS-CoV2 [8, 62, 97, 101, 110, 334]. Moreover, reduced TLR3 expression in peripheral blood has been linked to poor outcomes in severe COVID-19 patients [208].

TLR7 and TLR8 have been shown to sense ssRNA from viruses such as CVB, DENV, EV71, HCV, human immunodeficiency virus (HIV), IAV, vesicular stomatitis virus (VSV), and SARS-CoV [9, 64, 184, 193, 254, 296, 312, 335]. Genetic polymorphisms in the TLR7 and TLR8 genes have been related to susceptibility to HCV infection. Additionally, TLR7 SNPs have been associated with the severity of HIV and SARS-CoV2 infections [8, 225, 310]. Several studies have suggested that a loss of function in the X-chromosomal TLR7 could be a genetic factor in COVID-19 severity in males [3, 277, 281]. TLR9 provides protective immunity against various DNA viruses, including HSV, MCMV, HCMV, and poxviruses. HSV-1 and HSV-2 both activate TLR9, leading to the production of IFN-α and inflammatory cytokines from pDCs [159, 192]. MCMV is widely used as a model of HCMV in animal studies. Sensing MCMV by TLR9 has been shown to protect mice, as the mortality rate in TLR9 knockout mice was drastically increased after MCMV infection. In addition, NK cell activation and cytokine production, including IL-12 and IFNs were impaired in these mice [283]. TLR9 and TLR7 can differentially and cooperatively respond to MCMV infection. In TLR7/TLR9 double-knockout mice, MCMV infection resulted in reduced IFN levels, increased viral loads, and higher mortality compared to TLR7 or TLR9 single-knockout and wild-type mice. In MCMV-infected pDCs, IL-12p70 and IFN production were primarily TLR9-dependent, while TLR7 mediated the production of IFNs and TNFα [339]. Ectromelia virus (ECTV), the causative agent of mousepox, has been shown to rely on TLR9 for host defense, as TLR9-deficient mice exhibited drastically increased susceptibility to infection [255]. In addition to its role in host defense against these DNA viruses, TLR9 was also reported to detect DNA from varicella-zoster virus (VZV), Merkel cell polyomavirus, KSHV, EBV, and HIV [22, 84, 113, 255, 269, 316, 337]. In humans, TLR9 SNPs have been associated with an increased risk of HCMV disease in children and the rapid progression of HIV infection [26, 233].

## Anti-tumor and pro-tumor roles of NA-sensing TLR activations

**Fig. 2:**
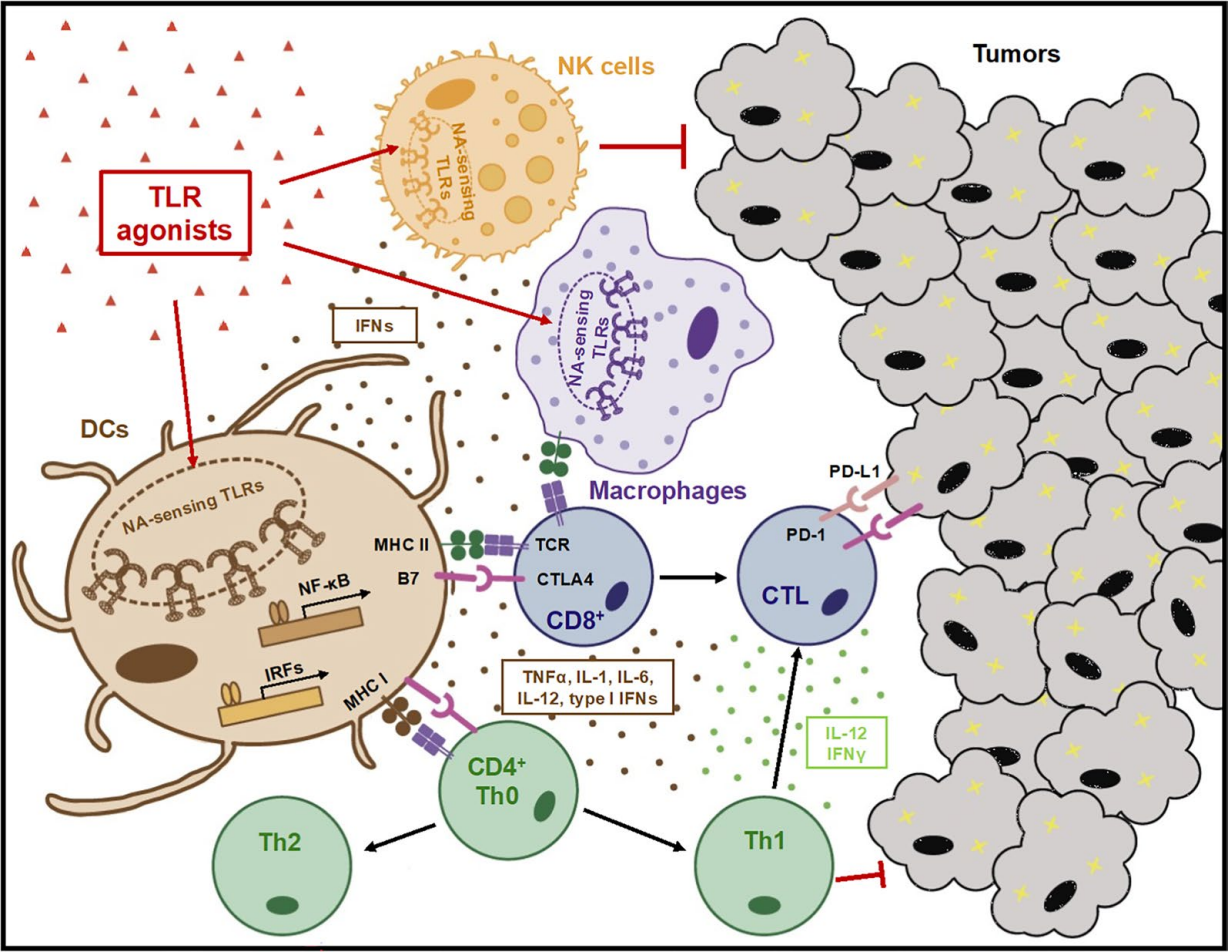
Roles of NA-sensing TLRs in anti-tumor strategies. Activation of NA-sensing TLRs triggers dendritic cells (DCs) and other immune cells to secrete proinflammatory cytokines, including TNFα, IL-1, IL-6, IL-12, and type I IFNs. Type I IFNs, in turn, activate natural killer (NK) cells and macrophages. While NK cells exert direct cytotoxicity against tumor cells, DCs and macrophages contribute to anti-tumor immunity through antigen presentation and IFN-γ secretion. NA-sensing TLR activation also enhances innate immune responses and upregulates the expression of co-stimulatory molecules on antigen-presenting cells (APCs), promoting effective antigen presentation to naïve T cells and supporting their differentiation. In conjunction with antigen presentation, IL-12 and IFN-γ secreted by innate immune cells drive CD4^+^ T cell polarization and the expansion of antigen-specific CD8^+^ T cell, contributing to robust anti-tumor responses and cancer cell eradication. These mechanisms highlight NA-sensing TLRs as potential therapeutic targets in cancer immunotherapy

The anti-tumor immune responses elicited by NA-sensing TLRs can be divided into two phases: the innate immune and the adaptive immune responses. TLR agonists activate an early antigen-independent innate immune response, which primes the subsequent adaptive immune response [11, 302]. During the initial innate immune phase, DCs, macrophages, and NK cells are activated. These innate immune cells produce cytokines and present antigens to activate adaptive immune cells. DCs produce inflammatory cytokines such as TNFα, IL-1, IL-6, and IL-12 and type I IFNs through the activation of NF-κB and IRFs. In the tumor microenvironment, macrophages are the major source of inflammatory cytokines. Both macrophages and NK cells are activated by IFNs produced by DCs. NK cells directly kill tumor cells during TLR activations-induced antitumor response, while DCs and macrophages function as major antigen-presenting cells (APCs) and sources of IFN-γ. These early TLR-activated innate immune responses are crucial for establishing antigen-specific adaptive antitumor immunity in the second phase. In the adaptive immune phase, activated APCs mature, increasing the expression of costimulatory molecules such as CD80, CD86, and MHC molecules, thereby enhancing antigen presentation to naïve T cells and promoting their differentiation. This antigen presentation, along with cytokines produced by innate immune cells-particularly IL-12 and IFN-γ-cooperatively drives the polarization of CD4^+^ T cells toward a T helper 1 (Th1) phenotype, which in turn promotes the expansion of antigen-specific CD8^+^ T cells [28, 33, 67, 130, 304]. Because these immune responses facilitate the eradication of cancer cells, NA-sensing TLRs represent promising targets for the development of anti-tumor therapies (Fig. 2).

While the intense immune response triggered by NA-sensing TLRs exhibits anti-tumoral activity, chronic inflammation elicited by these TLRs can sometimes promote tumor development [56, 325]. Chronic inflammation contributes to tumor formation and progression through several mechanisms. It can lead to the production of reactive oxygen species (ROSs), resulting in DNA damage, mutations, and increased cell proliferation. Additionally, chronic inflammation can induce matrix metalloproteinases (MMPs) and other enzymes involved in angiogenesis and tissue remodeling to support tumor growth and metastasis. Prolonged inflammation can also create an immunosuppressive tumor microenvironment by accumulating suppressive immune cells like M2 macrophages and Treg cells, which hinder immune responses that would otherwise target and eliminate cancer cells. NA-sensing TLRs play a pivotal role in tumor-associated inflammation. Both microbial components and host-derived molecules from dead cells caused by persistent irritants contribute to TLR-mediated inflammation [12, 56, 89, 118, 133, 147, 190, 242, 325, 326]. TLR3 overexpression in prostate carcinoma and oral cancer is associated with poor clinical outcomes [100, 332]. Similarly, TLR3 expression is elevated in gastric cancer and is significantly associated with reduced overall survival in patients with resectable tumors [75, 100, 332]. In multiple myeloma and chronic lymphocytic leukemia (CLL), TLR7 activation promotes tumor cell survival, proliferation, and the secretion of pro-inflammatory cytokines [182]. In lung cancer cells, TLR7 and TLR8 expressions enhance tumor cell survival and resistance to chemoresistance [43]. Pancreatic cancer progression is accelerated by TLR7 and TLR8 stimulation [105, 223, 228]. High TLR7 and TLR8 expression in CD133^+^ cancer stem cells in colorectal cancer is also associated with poor prognosis [104]. Similarly, TLR9 overexpression has been associated with increased inflammatory responses and poor prognosis in various cancers, including glioblastoma, prostate carcinoma, breast cancer, ovarian cancer, colorectal carcinoma, and HCC [23, 69, 93, 179, 189, 194, 303]. TLR9 is also highly expressed in gastric cancer, where it mediates *Helicobacter pylori*-induced inflammation, promoting gastric tumorigenesis [83, 289].

## Roles of NA-sensing TLRs in inflammatory autoimmune diseases

**Fig. 3:**
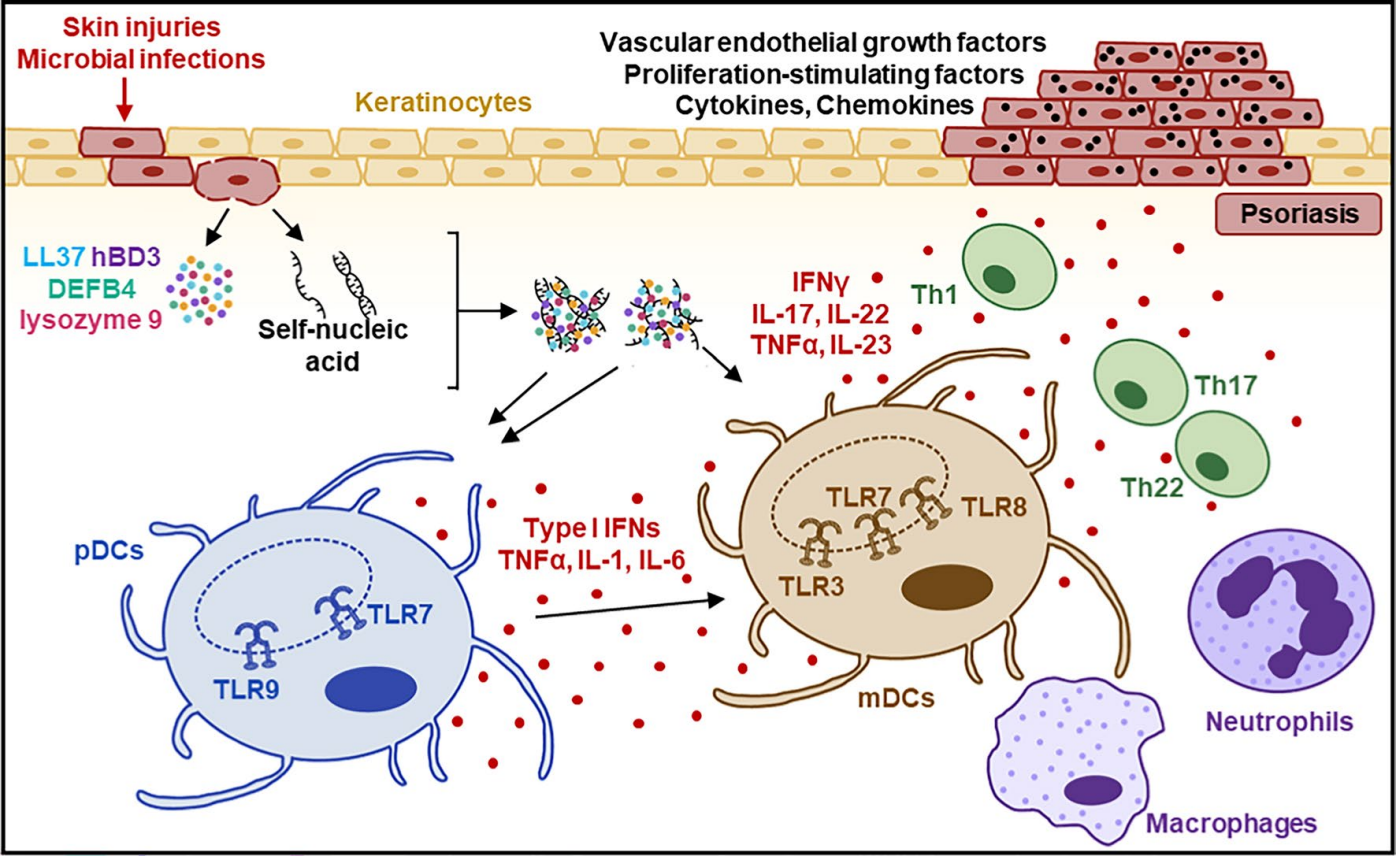
Roles of NA-sensing TLRs in psoriatic inflammation. Damaged keratinocytes release antimicrobial peptides, such as LL-37, alongside self-DNA and self-RNA, which forms LL-37/self-nucleic acid complexes that activate NA-sensing TLRs in plasmacytoid dendritic cells (pDCs) and myeloid dendritic cells (mDCs). These DCs secrete cytokines, including TNF-α, IL-1, IL-6, IL-12, IL-23, and type I interferons (IFNs), which promote the maturation and differentiation of Th1 and Th17 cells. The resulting cytokines, particularly IL-17, further stimulate keratinocytes, amplifying the inflammatory response characteristic of psoriasis

Psoriasis, a common inflammatory skin disorder, is recognized as an autoimmune disease. It is characterized by the abnormal activation of innate immune cells and pathogenic T cells, leading to skin inflammation and the hyperproliferation of keratinocytes, which result in the formation of erythematous scaly plaques [191, 200, 236]. The molecular mechanisms driving psoriasis pathogenesis involve the activation of NA-sensing TLRs in innate immune cells. During the initiation phase, external triggers like microbial infections and skin injuries prompt the release of nucleic acid-binding proteins, including antimicrobial peptides like LL-37 (also known as hCAP18, the 18 kDa human cationic antimicrobial protein), DEFB4 (β-defensin 4), hBD3 (human β-defensin 3), and lysozyme 9, from keratinocytes. Simultaneously, dying cells release self-DNA and self-RNA, which form complexes with these anti-microbial peptides, facilitating their entry into cells and activating NA-sensing TLRs [38, 92, 264]. Given the distinct expression of NA-sensing TLRs in different DC subsets, self-RNA, and self-DNA complexes activate TLR7 and TLR9 in pDCs, leading to the production of proinflammatory cytokines including TNFα, IL-1, IL-6, and type I IFNs [38, 92, 142, 151, 215, 264]. These cytokines further activate mDCs. In addition, self-RNA complexes can directly activate mDCs, inducing IL-12 and IL-23 production at psoriatic inflammatory sites. These cytokines drive the proliferation of T cells into Th1, Th17, and Th22 subsets, which in turn release additional cytokines. The production of IL-17, in synergy with other cytokines like TNFα and IL-22, induces keratinocyte activation, contributing to the development of the psoriasis phenotype. This leads to the amplification phase, where there is a broader activation of T-cell subsets and a buildup of psoriatic responses (Fig. 3). This phase is characterized by keratinocyte proliferation and the increased production of cytokines, chemokines, growth factors, and vascular endothelial growth factors at psoriatic sites [68, 196]. Psoriatic skin shows increased expression of LL-37, acting as an auto-antigen for activated T cells [164, 210, 218]. LL-37 also interacts with TLR9 and TLR7/TLR8 on DCs, further influencing the inflammatory response in psoriasis. Supporting the role of NA-sensing TLRs in psoriasis, genetic studies, including genome-wide association studies (GWAS), have identified associations between specific gene variants related to these TLRs and their signaling and effector molecules with psoriasis susceptibility and severity [224, 331].

**Fig. 4:**
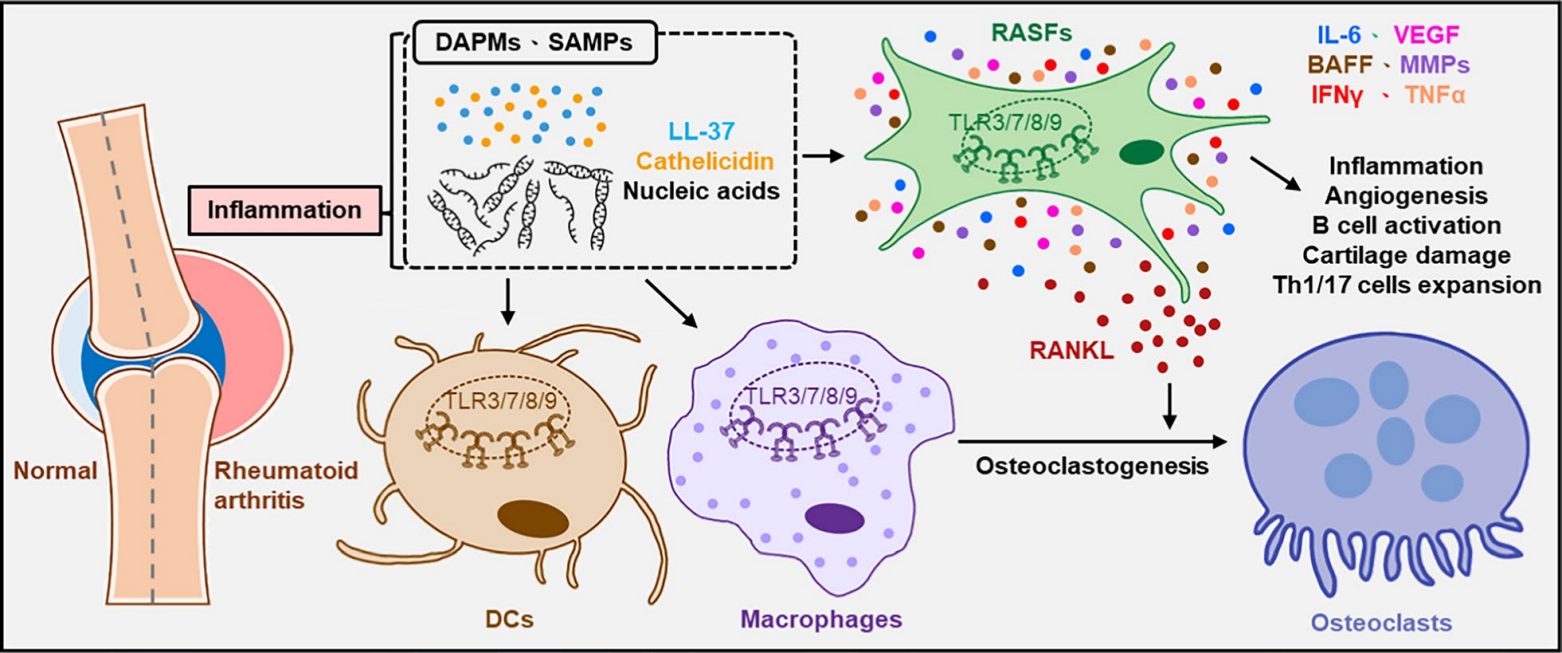
Roles of NA-sensing TLRs in the pathogenesis of rheumatoid arthritis. LL-37, along with self-DNA and self-RNA released from damaged cells, forms complexes that activate NA-sensing TLRs in synovial fibroblasts, macrophages, and DCs. This activation increases the production of pro-inflammatory cytokines within the synovium, promoting the differentiation of T cells into Th1 and Th17 subtypes and driving the maturation of macrophages into osteoclasts. These processes contribute to bone erosion and cartilage destruction in rheumatoid arthritis

Rheumatoid arthritis (RA) is a complex autoimmune disorder characterized by chronic inflammation that primarily affects the synovial joints, leading to progressive damage to both cartilage and bone. This condition is marked by the infiltration of inflammatory cells and the proliferation of fibroblasts within the synovial tissue, resulting in sustained inflammation and gradual joint destruction. In RA-afflicted joints, there are elevated levels of inflammatory mediators including metalloproteases (MMPs), vascular endothelial growth factor (VEGF), and a variety of pro-inflammatory cytokines. Macrophages play a central role in synovitis by producing cytokines to further promote inflammation. In addition, they activate RA synovial fibroblasts (RASF), which further produce cytokines, resulting in sustained inflammation. RASF also secrete receptor activators of nuclear factor κB ligand (RANKL), which promotes pathological bone erosion by facilitating the differentiation of monocytes/macrophages into osteoclasts [85, 109, 276]. NA-sensing TLR activations have been implicated in RA progression. Numerous studies have demonstrated aberrant expression of TLR3, 7, 8, and 9 within the synovial tissue of RA patients, with elevated levels observed particularly in RASFs, DCs, and macrophages [2, 49, 131, 163, 249]. Necrotic cell components, including RNA and DNA released from damaged cells, serve as endogenous ligands for these TLRs, activating the inflammatory cascade within the synovium. The presence of cathelicidin and LL-37 in RA synovial fluid protects nucleic acids from degradation, further perpetuating TLR-mediated inflammation. Upon activation of TLR3 by the released RNA, RASFs produce IL-6, MMPs, B cell activating factor (BAFF), and VEGF. These factors contribute to inflammation, cartilage damage, angiogenesis, and B cell activation, and can enhance the expansion of Th1 and Th17 cells [27, 131]. Additionally, TLR3 directly and indirectly promotes osteoclastogenesis in the RA synovium. TLR3 activation in monocytes increases their differentiation into osteoclasts, while activation in RASFs induces RANKL expression, further promoting osteoclasts differentiation [154] (Fig. 4). In a Danish population, TLR3 (rs3775291) SNP has been associated with the disease activities of seronegative RA [165]. Both TLR7 and TLR8 are expressed at higher levels in RA synovial macrophages and peripheral blood monocytes, with TLR8 expression notably elevated in the synovial tissue of RA patients [2]. RA patients carrying an M1V variant of TLR8 show reduced disease severity, as this variant leads to reduced production of inflammatory cytokines in response to ligand stimulation [295]. However, another report suggests that in RA monocytes, the expression of TLR7, but not TLR8, is associated with TNFα levels and disease activity score (DAS28). Single-strand RNA present in RA synovial fluid can induce TLR7-mediated transcription of TNFα in monocytes [37]. Moreover, small extracellular vesicles isolated from the synovial fluid of RA patients significantly enhance osteoclast differentiation. The extracellular vesicles contain miR-574-5p, which activates TLR7/8 signaling [119]. The TLR8 rs5741883 variant has been associated with the clinical characteristics of RA [73, 175, 176]. As a receptor for unmethylated CpG motifs containing DNA, TLR9 is also implicated in RA inflammation. Studies have revealed increased levels of cell-free DNA in the serum and articular cavities of RA patients compared to healthy individuals. This DNA can participate in the pathogenesis of RA through the TLR9 pathway [114, 115, 152, 251]. The TLR9 rs187084 variant increases susceptibility to RA in populations from Turkey and Poland, although this association has not yet been found in other populations [50, 73, 77, 96]. Additionally, a study involving RA patients receiving TNFα inhibitors treatment showed an association of TLR9 polymorphism (rs352139) with disease remission in these patients [155].

**Fig. 5:**
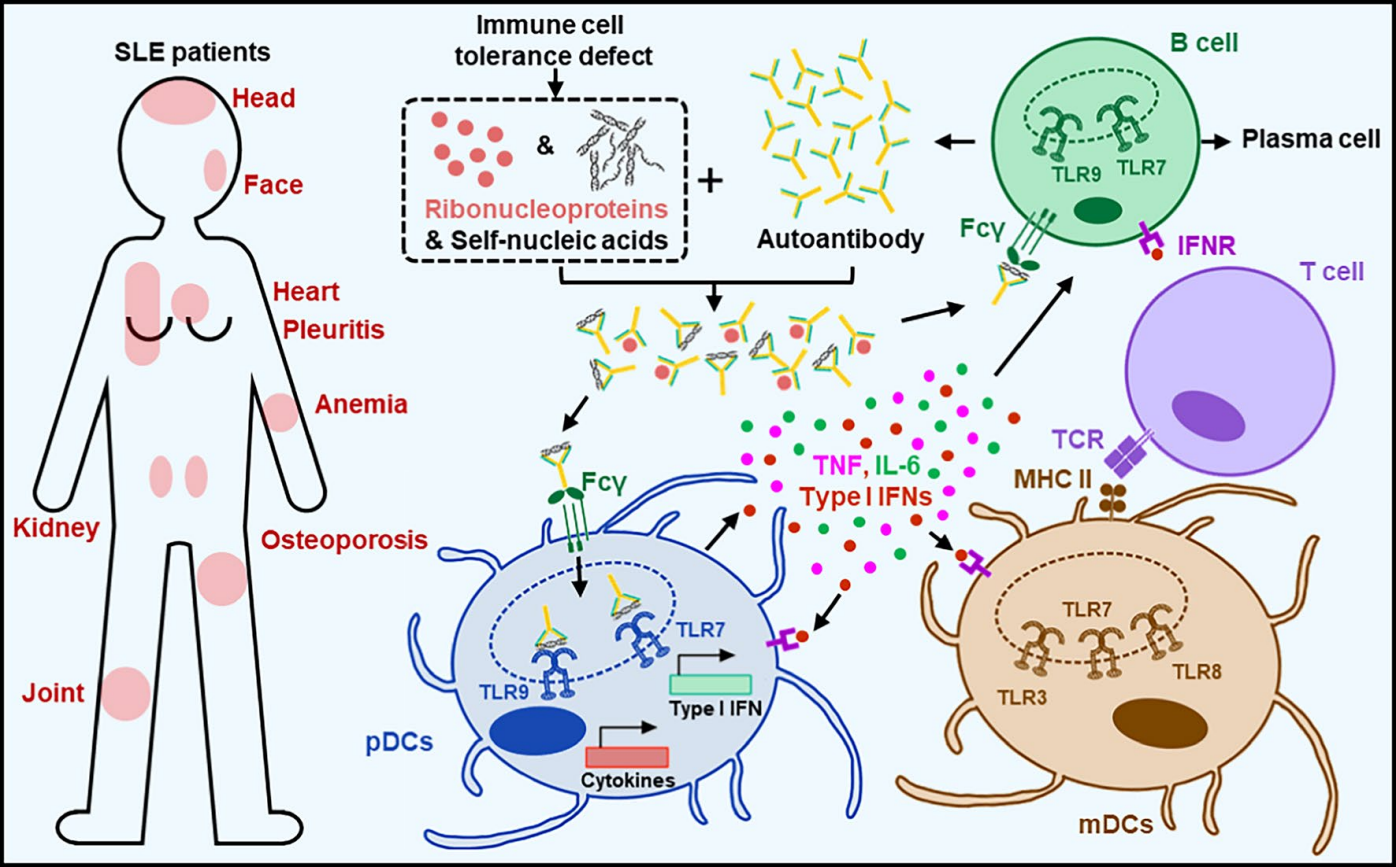
Roles of NA-sensing TLRs in the inflammatory response to self-nucleic acids in systemic lupus erythematosus. In patients with systemic lupus erythematosus (SLE), the loss of immune tolerance results in the production of autoantibodies that target self-nucleic acids and ribonucleoproteins released from dying cells. These nucleic acid-containing immune complexes bind to Fcγ receptors on cell surfaces and are subsequently internalized, where they interact with NA-sensing TLRs. Activation of these TLRs induces the production of pro-inflammatory cytokines and type I IFNs while upregulating the expression of antigen-presenting and co-stimulatory molecules. This enhances immune responses, including the activation of autoreactive B and T cells, which further produce autoantibodies. Consequently, this cycle perpetuates the autoimmune response in SLE

Systemic lupus erythematosus (SLE) is an autoimmune disease characterized by widespread inflammation and tissue damage affecting multiple organs and tissues, including the joints, skin, connective tissues, lungs, kidneys, blood vessels, and central nervous system. Although the exact pathophysiology of SLE is not yet fully clear, the disease is marked by elevated levels of type I IFNs and autoantibodies, which serve as both pathogenic drivers and diagnostic markers [98, 148, 273]. The prominent involvement of type I IFNs in the pathogenesis to SLE suggests that it belongs to a group of autoinflammatory diseases known as type I interferonopathies. These disorders are characterized by excessive type I IFN production, often driven by NA-sensing mechanism, including TLR7 [207, 301]. In SLE patients, immune cells lose tolerance to self-nucleic antigens, including self-nucleic acids and ribonucleoproteins released from dead cells. This loss of tolerance leads to the production of autoantibodies that specifically bind to these antigens, resulting in the formation of nucleic acid-containing immune complexes. These complexes are recognized by Fcγ receptors and TLRs, which repetitively stimulate inflammatory responses in immune cells [29, 167, 204, 307, 308] (Fig. 5). Numerous studies have shown association of SNPs with NA-sensing TLRs with SLE across different populations. Notable associations include those in TLR3 (rs3775296 and rs3775291), TLR7 (rs179008, rs3853839, and rs179010), TLR8 (rs3764879, rs3764880), and TLR9 (rs351240, rs352143, rs187084, and rs352139) [61, 72, 166, 177, 268, 311, 329]. Research has increasingly focused on the roles of TLR7 and TLR9 in the pathogenesis of SLE. The SLE-promoting effects of these TLRs arise from their direct activation in TLR-bearing cells [32]. Upon activation by nucleic acid-containing immune complexes, these TLRs trigger the production of proinflammatory cytokines and type I IFNs, promoting immune responses that include the activation of autoreactive B and T cells [204, 260, 308]. B cells express both TLR7 and TLR9, which perform multiple roles in SLE, including presenting antigens to T cells and activating myeloid cells to produce cytokines. In autoreactive B cells, TLR7 activation provides the required co-stimulation for proliferation and differentiation into plasma cells, resulting in the production of autoantibodies. These autoreactive B cells produce autoantibodies with the cooperative help of autoreactive T cells [95, 135, 279]. In animal model studies of SLE, TLR7 has been shown to have an inflammatory role, enhancing the autoimmune response, whereas TLR9 appears to have a protective role, as studies showed increased severity in TLR9-deficient animals [46, 256, 293, 320, 327]. One proposed mechanism for the protective function of TLR9 is its antagonistic effect on the intracellular trafficking of TLR7 from the ER to endosomes for activation. TLR9 competes with TLR7 for binding to UNC93B1 with a higher affinity, thereby inhibiting TLR7 function [90, 91]. Additionally, overexpression of TLR7 in mice leads to the proliferation of immature transitional B cells and hinders the clearance of autoreactive B cells, which aligns with the increased number of transitional B cells found in many SLE patients [99, 274]. In contrast, TLR9 supports immune tolerance. Although TLR9 ligands activate B cells, simultaneous engagement of the B-cell receptor (BCR) and TLR9—mimicking the activation of autoreactive B cells by self-antigens—triggers cell death via apoptosis after a brief expansion of B cells, thereby preventing the production of anti-dsDNA autoantibodies. Thus, TLR9 may play a critical role in restricting the activation of autoreactive B cells [220, 275]. The recent discovery of a TLR7 gain-of-function mutation (Y264H) in patients with childhood SLE further provided strong evidence for the role of TLR7 activation in the disease’s pathogenesis. This specific mutation, which affects the TLR7 ligand-binding site, significantly increases the receptor’s affinity for its ligands. When this Y264H variant was introduced into mice, they spontaneously developed lupus-like symptoms. Remarkably, these lupus-like phenotypes were reversed when the mice were crossed with MyD88-knockout mice, highlighting the essential role of TLR7-MyD88 signaling in lupus development [30]. The TLR7 is located on the X chromosome [47]. In females, one X chromosome is typically inactivated in each cell, but up to 30% of X-linked genes, including TLR7, can escape this inactivation. This biallelic expression results in higher TLR7 activity in females, contributing to their increased susceptibility to SLE. Recent studies confirm that this escape from X chromosome inactivation leads to heightened TLR7 expression and responsiveness in female immune cells, which may help explain why women are disproportionately affected by SLE [111, 280].

## Targeting NA-sensing TLRs for drug development

The activation of NA-sensing TLRs induces cytokine production and increases antigen presentation, thereby bridging innate and adaptive immunity, and enhancing both antigen-independent and antigen-dependent immune responses. As a result, various NA-sensing TLR agonists have been developed as antiviral therapeutic agents and vaccine adjuvants [153, 267, 322]. Additionally, activation of NA-sensing TLRs enhances cytotoxic T cell-mediated tumor killing, leading to the investigation of these agonists for cancer therapy [36, 169, 250] (Table 1). While these agonists are being explored for their therapeutic applications, NA-sensing TLR antagonists are being investigated as treatments for inflammatory autoimmune diseases [18, 82, 234] (Table 2).

**Table 1:** NA-sensing TLR agonists under clinical investigation

TLR	TLR Agonist	Indication	Clinical stage	Status	Trial number
TLR3	Ampligen	HIV	Phase I/II	Completed	NCT02071095
		HIV vaccine	Phase I	Completed	NCT01127464
	Poly-ICLC (Hiltonol, NEO-PV-01, PGV001)	COVID-19	Phase I	Completed	NCT04672291
		COVID-19	Phase I	Completed	NCT04672291
		Influenza, Severe Acute Respiratory Distress Syndrome, Smallpox, Ebola, Unknown Respiratory Viruses	Phase I	Completed	NCT00646152
		Nonsquamous Non-small Cell Lung Cancer	Phase I	Completed	NCT03380871
		Solid Tumors	Phase I		NCT02721043
		Malignant Melanoma, Non-Small Cell Lung Carcinoma, Urinary Bladder Cancer	Phase I	Completed	NCT02897765
		Melanoma	Phase I/II	Completed	NCT01079741
	Poly I:Poly C_12_U (Rintatolimod)	Influenza	Phase I/II	Terminated	NCT01591473
		Breast Cancer	Phase I/II	Recruiting	NCT05756166
		Peritoneal Surface Malignancies	Phase I/II	Completed	NCT02151448
		Colorectal Cancer	Phase II	Completed	NCT03403634
		Pancreatic Cancer	Phase II	Recruiting	NCT05494697
		HLA-A2 Positive Refractory Melanoma	Phase II	Recruiting	NCT04093323
	PIKA	Rabies	Phase II	Completed	NCT02956421
TLR4	GLA-LSQ	HIV	Phase I	Completed	NCT04177355
	GLA-AF	Hookworm	Phase II	Completed	NCT03172975
TLR7	Vesatolimod	Chronic Hepatitis B	Phase II	Completed	NCT02166047
		Chronic Hepatitis B	Phase II	Completed	NCT02579382
		HIV-1	Phase I	Completed	NCT03060447
		HIV-1	Phase I/II	Recruiting	NCT06071767
		HIV-1	Phase II	Completed	NCT05281510
	RO6864018	Chronic Hepatitis B	Phase II	Completed	NCT02391805
	RO7020531	Chronic Hepatitis B	Phase I	Completed	NCT02956850
	RO7119929	Hepatocellular Carcinoma, Biliary Tract Cancer, Secondary Liver Cancer	Phase I	Completed	NCT04338685
	APR003	Advanced Colorectal Carcinoma	Phase I	Completed	NCT04645797
	LHC165	Solid Tumors	Phase I	Terminated	NCT03301896
	TQ-A3334	Non-small Cell Lung Cancer	Phase I/II	Recruiting	NCT04273815
	SHR2150	Unresectable/Metastatic Solid Tumors	Phase I/II	Recruiting	NCT04588324
	DSP-0509	Advanced Solid Tumors	Phase I/II	Terminated	NCT03416335
	BNT411	Solid Tumor, Extensive-stage Small Cell Lung Cancer	Phase I/II	Terminated	NCT04101357
TLR7/8	Resiquimod	Influenza Vaccine	Phase I	Completed	NCT01737580
		Hepatitis B	Phase I/II	Completed	NCT00175435
	3 M-052-AF	HIV	Phase I	Completed	NCT04177355
		Zika Virus	Phase I	Recruiting	NCT06334393
	CV8102	Rabies	Phase I	Completed	NCT02238756
		Advanced Melanoma, Squamous Cell Carcinoma of the Skin, Squamous Cell Carcinoma of the Head and Neck, Adenoid Cystic Carcinoma	Phase I	Active, not recruiting	NCT03291002
	BDB001	Advanced Solid Tumors	Phase II	Recruiting	NCT03915678
		Solid Tumors	Phase II	Completed	NCT04819373
	BDB018	Advanced Solid Tumors	Phase I	Active, not recruiting	NCT04840394
	TransCon	Solid Tumors	Phase I/II	Active, not recruiting	NCT04799054
	BDC-1001	HER2-Expressing Solid Tumors	Phase I/II	Terminated	NCT04278144
TLR7/9	IMO-3100	Plaque Psoriasis	Phase II	Completed	NCT01622348
TLR8	SBT6050	HER2 Positive Solid Tumors	Phase I	Active, not recruiting	NCT04460456
	VTX-2337	Ovarian Clear Cell Cystadenocarcinoma	Phase I	Completed	NCT01294293
	GS-9688 (Selgantolimod)	Chronic Hepatitis B	Phase II	Completed	NCT03615066
		Squamous Cell Cancer of Head and Neck	Phase I	Completed	NCT01334177
		Squamous Cell Cancer of Head and Neck	Phase II	Completed	NCT01836029
		Epithelial Ovarian Cancer, Fallopian Tube Cancer, Primary Peritoneal Cancer	Phase II	Completed	NCT01666444
TLR9	CMP-001 (Vidutolimod)	Advanced Melanoma	Phase I	Completed	NCT02680184
		Radiosurgery for Liver Metastases in Colorectal Carcinoma	Phase I	Completed	NCT03507699
		Stage IV Pancreatic and Other Cancers Except Melanoma	Phase I/II	Terminated	NCT04387071
		Advanced Melanoma	Phase II	Terminated	NCT04698187
		Melanoma	Phase II	Completed	NCT04401995
		Squamous Cell Carcinoma of Head and Neck	Phase II	Terminated	NCT04633278
		Melanoma, Lymph Node Cancer	Phase II	Completed	NCT03618641
	SD-101	Pancreatic Adenocarcinoma	Phase I	Completed	NCT04050085
		Solid Neoplasm	Phase I	Completed	NCT03831295
		Metastatic Uveal Melanoma	Phase I	Active, not recruiting	NCT04935229
		Hepatocellular Carcinoma (HCC), Intrahepatic Cholangiocarcinoma (ICC)	Phase I/II	Active, not recruiting	NCT05220722
		Breast Cancer	Phase II	Recruiting	NCT01042379
		Prostatic Neoplasms	Phase II	Active, not recruiting	NCT03007732
	MGN1703 (Lefitolimod)	Melanoma	Phase I	Active, not recruiting	NCT02668770
		HIV-1	Phase I/II	Completed	NCT02443935
		HIV-1-infection	Phase II	Completed	NCT03837756
	Tilsotolimod	Advanced Solid Tumors Cancer	Phase I	Completed	NCT04196283
		Advanced Cancer	Phase I	Terminated	NCT04270864
		Melanoma	Phase II	Recruiting	NCT04126876
		Solid Tumors	Phase II	Completed	NCT03865082
	CpG7909	Influenza	Phase I	Completed	NCT00559975
		Bacillus Anthracis (Anthrax)	Phase I	Completed	NCT01263691
		Solid tumors harboring KRAS mutations	Phase I	Active, not recruiting	NCT04853017
		Solid tumors with mutated KRAS/NRAS	Phase I/II	Active, not recruiting	NCT05726864
	CpG7909-ODN (VaxImmune)	Plasmodium Falciparum Malaria	Phase I	Completed	NCT00344539
		HIV, Hepatitis B	Phase I/II	Completed	NCT00100633
	CpG 1018	HIV	Phase I	Completed	NCT04177355
		Zika Virus	Phase I	Recruiting	NCT06334393
	CPG 10104	Hookworm	Phase II	Completed	NCT03172975
	IMO-2125	Hepatitis C	Phase I	Completed	NCT00728936
	IC31	Tuberculosis	Phase II	Active, not recruiting	NCT03512249
	PF-3512676	Mantle cell lymphoma	Phase II	Completed	NCT00490529

*COVID-19* Coronavirus Disease 2019, *HER2* Human Epidermal Growth Factor Receptor 2, *HIV* Human Immunodeficiency Virus, *HLA* Histocompatibility Leukocyte Antigen, *KRAS* Kristen Rat Sarcoma Viral Oncogene Homolog, *NRAS* Neuroblastoma RAS Viral Oncogene Homolog, *PIKA* Polyinosinic-Polycytidylic Acid Based Adjuvant, *Poly-ICLC* Polyinosinic-Polycytidylic Acid with Polylysine and Carboxymethylcellulose, *TLR* Toll-like Receptor

**Table 2:** NA-sensing TLR antagonists under clinical investigation

TLR	TLR Antagonist	Indication	Clinical stage	Status	Trial number
TLR7	BAY1834845	Psoriasis	Phase I	Completed	NCT03493269
	PF-06650833	Healthy	Phase I	Completed	NCT02485769
		Rheumatoid Arthritis	Phase II	Completed	NCT02996500
TLR7/8	E6742	Lupus Erythematosus, Systemic	Phase I/II	Completed	NCT05278663
	Afimetoran (BMS 986256)	Systemic Lupus Erythematosus	Phase II	Recruiting	NCT04895696
	Enpatoran	Systemic Lupus Erythematosus	Phase II	Completed	NCT05162586
TLR7/9	Hydroxychloroquine (HCQ)	Systemic Lupus Erythematosus	Not applicable	Completed	NCT02842814
		Rheumatoid Arthritis	Phase IV	Completed	NCT03855007
TLR7/8/9	IMO-8400	Plaque Psoriasis	Phase II	Completed	NCT01899729
TLR9	CPG-52364	Healthy	Phase I	Completed	NCT00547014

## Antiviral and vaccine adjuvant functions of NA-sensing TLR agonists

Imiquimod, a TLR7 agonist, has been FDA-approved for the treatment of external genital and perianal warts caused by the human papillomavirus (HPV) infection [294, 309]. In addition, several NA-sensing TLR agonists have undergone clinical investigation for the treatment of viral infections. Poly(I:C), a synthetic dsRNA that activates TLR3, has been chemically modified into poly(I:C_12_U) (Ampligen, Rintatolimod, Hemisphrex) by adding mismatched uracil and guanine bases at specific intervals along the RNA chain. This TLR3 agonist has been investigated as a therapeutic agent against HIV. Another formulation, Poly-ICLC (Hiltonol, Oncovir), a poly-l-lysine carboxymethylcellulose-stabilized poly(I:C), has also been evaluated in clinical trials for HIV infection (NCT00000735, NCT02071095), and its nasal form has been investigated for the prevention of COVID-19 and other respiratory viral Infections (NCT04672291, NCT00646152) [1, 76, 203, 261]. Vesatolimod (GS-9620), an oral TLR7 agonist, is currently under investigation for the treatment of HBV and HIV infections. RO7020531 (also called RG7854), and RO6870868, oral prodrugs of the TLR7 agonist, are being explored for chronic HBV infection. Additionally, RO6864018, a double prodrug, is converted by nonspecific esterases to RO6870868 and then to an active TLR7‐specific agonist by aldehyde oxidase. This compound has been studied in patients with chronic HBV and HCV infections (NCT02166047, NCT03060447, NCT02391805, NCT02956850) [5, 106, 203, 247, 328]. TLR9 agonists, such as MGN1703 (Lefitolimod) and IMO-2125 (Tilsotolimod), have also been evaluated for HIV and HCV treatments, respectively (NCT02443935, NCT00728936) [108, 248, 306]. In addition to monotherapy, there is growing interest in combining TLR agonists with other therapeutic approaches to enhance antiviral efficacy. For examples, Poly-ICLC has been evaluated in combination with combination antiretroviral therapy (cART) in HIV-infected patients to evaluate safety and viral suppression (NCT02071095) [203, 261]. Vesatolimod combined with tenofovir disoproxil fumarate has also been investigated for HBV infection (NCT02579382) [5]. Similiarly, selgantolimod, an oral TLR8 agonist, has been tested with tenofovir alafenamide for HBV treatment (NCT03615066) [139]. Two additional clinical trials have evaluated vesatolimod as an immunomodulator in combination with broadly neutralizing anti-HIV-1 antibodies (bNAbs) and a therapeutic vaccine for HIV remission (NCT05281510 and NCT06071767). Likewise, lefitolimod and bNAbs, both individually and in combination, are being tested for antiretroviral therapy (ART)-free virologic control in patients with HIV-1 on ART (NCT03837756) [108].

In the context of vaccine development, several NA-sensing TLR agonists have demonstrated effectiveness as adjuvants, enhancing the immunogenicity of various vaccines. Two TLR9 agonists and one TLR7/8 agonist have already been successfully incorporated into vaccines used in humans. CpG-1018, a TLR9 agonist absorbed onto aluminum salt, is included in an FDA-approved HBV vaccine and has also been used in emergency use authorized (EUA) COVID-19 vaccines to boost immune responses against viral infections. CpG-55.2, another TLR9 agonist, and imidazoquinolin, a TLR7/8 agonist, have also been formulated as adjuvants in EUA COVID-19 vaccines [39, 126, 136, 221, 322]. Several other NA-sensing TLR agonists have been clinically investigated as vaccine adjuvants. These include: TLR3 agonists: poly(I:C_12_U), poly-ICLC, and PIKA; TLR7/8 agonists: Resiquimod, 3M-052, CV8012, and Vesatolimod; TLR9 agonists: CpG-7907, CpG-10104, and IC31. Poly(I:C_12_U) was tested in earlier clinical studies as an adjuvant for a nasal influenza vaccine, while poly-ICLC is currently evaluated for safety in nasal delivery for COVID-19-vaccinated individuals. It has also been investigated as an adjuvant in phase I clinical trials for anti-HIV vaccines (NCT01591473, NCT04672291, NCT01127464) [13, 160, 231]. PIKA, a kanamycin- and calcium-stabilized formulation of poly(I:C), has been studied as an adjuvant for rabies and COVID-19 vaccines (NCT02956421, ACTRN12621001009808)[145, 188, 317]. Resiquimod (R848), an imidazoquinoline compound for activation of TLR7 and TLR8, has been tested as an adjuvant for IAV and HBV vaccines (NCT01737580, NCT00175435) [54, 74]. 3M-052, a structural analog of resiquimod, incorporates a hydrophobic fatty acyl chain to improve bioavailability at the immunization site of immunization and can be formulated into emulsions or liposomes. A combination of 3M-052 with aluminum salt has been investigated as an adjuvant for SARS-CoV-2 vaccine. Additionally, 3M-052-0AF, a water-soluble derivative of 3M-052, also absorbed on aluminum salt, is being evaluated for as an adjuvant for HIV, Zika, and SARS-CoV-2 vaccines (NCT04177355, NCT06334393) [112, 238]. CV8102, an RNA-based TLR7/8 agonist with additional activity on RIG-I, has been investigated for improving the immunogenicity of a licensed rabies vaccine (NCT02238756) [66]. CpG-7909 (also known as CpG-2006, PF-3512676, VaxImmune, ProMuneT, and Agatolimod) is the most thoroughly investigated TLR9 agonist. It has been tested in various vaccine candidates targeting malaria, influenza, anthrax, and HBV (NCT00344539, NCT00100633, NCT00559975, NCT01263691) [51–53, 70, 128, 313]. CpG-10104 has been studied as an adjuvant for a vaccine against hookworm infections, while IC31, a CpG-ODN-containing antimicrobial peptide, has been investigated as an adjuvant for pulmonary tuberculosis vaccines (NCT03172975, NCT03512249) [140, 285].

## Anti-tumor activity of NA-sensing TLR agonists

Among TLR3 agonists, poly(I:C_12_U) and poly-ICLC are actively being investigated in clinical studies for their anti-tumor properties. Poly(I:C_12_U) has been explored in combination therapies, such as with IFN-α2b and celecoxib, for metastatic colorectal cancer with liver metastasis, showing a favorable safety profile (NCT03403634) [216]. A current trial is recruiting patients for a combination treatment including poly(I:C_12_U), IFN-α2b, celecoxib, and pembrolizumab for metastatic triple-negative breast cancer. Additionally, a phase II trial investigating poly(I:C_12_U) in patients with locally advanced pancreatic adenocarcinoma following FOLFIRINOX treatment has begun enrolling participants. Other clinical studies focus on the adjuvant activity of poly-ICLC and poly(I:C_12_U) in cancer vaccines. In lung cancer, a poly-ICLC-adjuvanted personalized neoantigen vaccine (NEO-PV-01) combined with chemotherapy or anti-PD-1 therapy as first-line treatment for advanced non-squamous non-small cell lung cancer has shown good safety and immunogenicity (NCT05756166, NCT05494697, NCT03380871) [14, 15]. Early-phase studies also explored poly-ICLC-adjuvanted NY-ESO-1 protein vaccines combined with Montanide for melanoma, and with decitabine for myelodysplastic syndrome, both effectively activating T-cell immunity in patients (NCT01079741) [252]. Two completed phase I trials involving poly-ICLC-adjuvanted vaccines, PGV001 and NEO-PV-01 with nivolumab, reported favorable safety and immunogenicity (NCT02721043, NCT02897765) [201, 230]. Additionally, a study evaluated autologous α-type-1 polarized DC (αDC1) vaccines loaded with autologous tumor material and combined with poly(I:C_12_U), IFN-α2b, and celecoxib for the treatment of peritoneal surface malignancies, demonstrated the safety of this combination. However, a key challenge in this DC-based therapy is obtaining sufficient cancer cells to generate enough autologous tumor-pulsed αDC1 for immunotherapy (NCT02151448) [243]. A phase II study is currently recruiting patients to investigate this αDC1 combination approach in PD-1/PD-L1-resistant melanoma (NCT04093323).

In addition to imiquimod, which has been approved for the treatment of superficial basal cell carcinoma and actinic keratosis, several TLR7 agonists, including TQ-A3334, SHR2150, RO7119929, APR003, BNT411, DSP-0509, and LCH 165, are under investigation in clinical trials. Among these, TQ-A3334, SHR2150, RO7119929, and APR003 are designed for oral administration, BNT411 and DSP-0509 are administered intravenously, and LCH165 is given via intratumoral injection [88, 250]. These agents have been evaluated in early-phase trials, both as monotherapies and in combination with chemotherapy, biologics, or immune checkpoint inhibitors for the treatment of solid tumors (NCT04273815, NCT04588324, NCT04338685, NCT03416335, NCT04645797, NCT04101357, NCT03301896) [55, 209, 229, 278, 282, 324]. The TLR8 agonist VTX-2337 (Motolimod) has been studied in patients with ovarian and squamous cell cancers in combination with chemotherapy or immune checkpoint inhibitors (NCT01294293, NCT01666444, NCT01334177, NCT01836029) [45, 212, 213]. Additionally, a HER2 monoclonal antibody conjugated to a TLR8 agonist, sbt-6050, is being investigated in combination with pembrolizumab for HER2-positive solid tumors (NCT04460456) [71]. Several dual TLR7/8 agonists, including TransCon, BDC-1001, BDB001, BDB018, and CV8102, are also under clinical investigation. TransCon is a prodrug of resiquimod designed for intratumoral retention; BDC-1001 is a HER2 monoclonal antibody conjugate; and BDB018, a refined analog of BDB001, aims to enhance immunostimulatory activity while preserving the safety profile of its predecessor. CV8102 contains an RNA structure that activates both TLR7/8 and the RIG-I pathway. Like their TLR7 counterparts, these dual TLR7/8 agonists are being explored in early-phase trials either alone or in combination with other therapies for solid tumors (NCT04799054, NCT04278144, NCT03915678, NCT04819373, NCT04840394, NCT03291002) [181, 195, 237, 330].

Various CpG-ODNs have been explored as antitumor therapies in clinical trials [4, 206]. Among them, CpG-ODN 7909 (also known as CpG-2006, PF-3512676, or Agatolimod) is the most extensively studied. CpG-7909 has been tested as a monotherapy for melanoma, basal cell carcinoma, renal cell cancer, and cutaneous T-cell lymphoma, using subcutaneous, intravenous, and intratumoral routes. While it induces localized antitumor immune responses with minimal toxicity, its impact on tumor burden has been modest [156, 292]. As a result, research has shifted towards modifying the structure of TLR9 agonists to enhance their immune-stimulatory properties, and exploring their use in combination therapies with existing cancer treatments. For example, in a phase I/II clinical study, patients with mantle cell lymphoma in remission after immunochemotherapy were vaccinated with irradiated, CpG-7909-treated autologous tumor cells. The vaccine-primed lymphocytes were then collected and reinfused through autologous transplantation. This strategy proved feasible and safe (NCT00490529) [87]. Another TLR9 agonist, ELI-002, is a lipid-modified version of CpG-7909 designed to target lymph nodes. ELI-002 incorporates either two (ELI-002 2P) or seven (ELI-002 7P) KRAS-mutated Amph-peptides and is currently being investigated as a cancer vaccine in patients with solid tumors harboring KRAS mutations (NCT04853017, NCT05726864) [232].

Beyond CpG-7909, several other TLR9 agonists are under clinical investigation for cancer therapy, including MGN1703 (Lefitolimod), IMO-2125 (Tilsotolimod), SD-101, and CMP-001. MGN1703, a dumbbell-shaped, double-stranded DNA molecule, is being evaluated in combination with ipilimumab for patients with advanced solid tumors (NCT02668770) [245]. IMO-2125, a 3’-linked CpG dimer administered intratumorally, is being investigated as monotherapy and in combination with anti-OX40 mAb or immune checkpoint inhibitors for solid tumors (NCT04196283, NCT04126876, NCT03865082, NCT04270864) [16, 170, 219]. SD-101 has been investigated in several clinical trials across different cancer types. These include combinations with nivolumab and radiation therapy for metastatic pancreatic cancer, pembrolizumab as neoadjuvant therapy in breast cancer, and with pembrolizumab, androgen deprivation and radiation therapy for oligometastatic prostate cancer (NCT04050085, NCT01042379, NCT03007732) [41, 80, 323]. Additional trials are assessing SD-101 in combination with nivolumab and ipilimumab in patients with uveal melanoma or liver tumors, as well as with BMS986178 in patients with solid tumors (NCT04935229, NCT05220722, NCT03831295) [127, 174, 214]. CMP-001, a CpG-ODN encapsulated in virus-like particles (VLPs) to enhance stability, is currently being evaluated as monotherapy and in combination with pembrolizumab in patients with PD-L1 inhibitor-refractory advanced melanoma. Ongoing studies are also investigating CMP-001 in combination with immune checkpoint inhibitors across several cancer types, including melanoma, recurrent/metastatic squamous cell carcinoma of the head and neck (SCCHN), metastatic colorectal cancer (CRC), metastatic pancreatic cancer, and other advanced malignancies. A recent phase II trial evaluated CMP-001 and nivolumab as neoadjuvant therapy in melanoma (NCT02680184, NCT04698187, NCT04401995, NCT04387071, NCT04633278, NCT03507699, NCT03618641) [58, 158, 168, 246, 319].

## NA-sensing TLR antagonists for treating inflammatory autoimmune diseases

Most NA-sensing TLR agonists under investigation act by directly targeting their respective receptors, while TLR antagonists function either by inhibiting the receptors themselves or by blocking downstream signaling pathways [234, 286]. Hydroxychloroquine, an antimalarial drug currently used to treat RA and SLE, is one such inhibitor of NA-sensing TLR signaling [65, 265]. Due to its lipophilic properties, hydroxychloroquine accumulates in lysosomes and endosomes, where it interferes with ligand binding to NA-sensing TLRs and inhibits downstream activation [162]. Hydroxychloroquine, in combination with glucocorticoid, has been clinically investigated for SLE (NCT02842814). In RA, Iguratimod, either alone or stepwise in combination with methotrexate, hydroxychloroquine, and prednisone, has been studied in clinical trials (NCT03855007). IMO-3100, a TLR7/9 antagonist, and IMO-8400, a TLR7/8/9 antagonist, block TLR activation by competing for receptor binding. These antagonists have been evaluated in clinical trials for moderate to severe psoriasis, with IMO-8400 demonstrating clinical activity in a phase IIa study (NCT01622348, NCT01899729) [17, 338]. IRAK4, a signaling molecule downstream of multiple TLRs, has also emerged as a therapeutic target. BAY-1834845 and BAY1830839, both IRAK4 inhibitors, have been investigated for safety and pharmacokinetics in healthy volunteers and psoriasis patients (NCT03493269) [141]. IRAK4 inhibitors are also being explored as treatments for RA. PF-06650833, an oral small-molecule IRAK4 inhibitor, showed favorable safety and pharmacokinetic profiles in phase I trials, and was subsequently evaluated in a phase IIb trial in RA patients with moderate to severe disease refractory to methotrexate (NCT02485769, NCT02996500) [57, 318].

Compared to psoriasis and RA, NA-sensing TLR inhibitors have been more extensively investigated for SLE [146, 288]. Several small-molecule inhibitors targeting TLR7, TLR8, and TLR9 are under study. GpG-52364, a triple TLR7/8/9 inhibitor, was evaluated in a phase I study for SLE but did not proceed to further development (NCT00547014) [240]. Among dual TLR7/8 inhibitors, the most promising candidates currently in clinical trials for SLE include Afimetoran (BMS-986256), Enpatoran (M5049), MHV370, and E6742. Afimetoran is undergoing a phase II study to assess safety and efficacy in SLE patients (NCT04895696, EudraCT 2019–004021-25) [244]. Enpatoran has been evaluated in a phase II study for both SLE and cutaneous lupus erythematosus (CLE) (NCT05162586) [239]. MHV370 was assessed in a phase I study and demonstrated good tolerability in healthy adults (EudraCT 2017-004559-21) [116, 271]. E6742 has been assessed in early-phase clinical trials, including a phase I/II study in SLE patients that showed a favorable safety profile and and promising efficacy, warranting further development for SLE treatment (NCT 05278663) [287]. Regarding inhibitors targeting TLR signaling, participants with SLE are being recruited for a phase IIa study to evaluate the safety and efficacy of Edecesertib (GS-5718), another IRAK4 inhibitor (NCT05629208) [10].

## Concluding remarks and future prospects

Since the discovery of NA-sensing TLRs two decades ago, research in this area has expanded significantly. These studies have uncovered the cellular signaling pathways and regulatory mechanisms that drive immune activation, highlighting the crucial role of NA-sensing TLRs in recognizing nucleic acids from pathogens and initiating essential immune responses. One of the most exciting developments in NA-sensing TLR research is the advancement of agonists for use as vaccine adjuvants and cancer immunotherapy. In addition to the approved applications for TLR7 and TLR9 agonists, numerous clinical trials are ongoing, further demonstrating the therapeutic potential of these agents. Another promising avenue is the investigation of NA-sensing TLRs in autoimmune and inflammatory diseases, with TLR7 and TLR8 receiving particular attention due to their X chromosome linkage and involvement in gender-biased diseases like SLE.

Despite these promising developments, several challenges remain. In cancer, NA-sensing TLR activation has dual and sometimes paradoxical effects. While it can enhance antitumor immunity by promoting DC maturation and type I IFN responses, it may also contribute to tumor progression by sustaining chronic inflammation and immune suppression. The tumor microenvironment often supports immunosuppressive cell types, such as myeloid-derived suppressor cells and tumor-associated macrophages, making it necessary to develop strategies that selectively enhance antitumor immunity while minimizing pro-tumor effects. To overcome this challenge, a growing trend in cancer therapy involves combining NA-sensing TLR agonists with other modalities. These TLR agonists can potentiate the effects of chemotherapy by activating immune pathways that recruit effector cells to the tumor site. When combined with immune checkpoint inhibitors, TLR agonists have been shown to further strengthen antitumor responses, enhancing the likelihood of tumor eradication. The rational integration of these agents with complementary therapies may improve efficacy, mitigate adverse effects, and enable personalized treatment strategies tailored to individual patient profiles.

Another significant challenge is the lack of receptor selectivity in many NA-based TLR agonists, which can lead to off-target immune activation, systemic inflammation, and cytokine release syndrome. These effects raise concerns about the potential for autoimmunity, as excessive TLR stimulation may disrupt immune tolerance, trigger inflammatory autoimmune diseases, or exacerbate tissue damage during viral infections. To address these risks, emerging precision-targeting strategies are being developed. These include chemical modifications to enhance agonist selectivity for specific TLRs, as well as nanoparticle-based delivery systems designed to improve targeting while minimizing unintended immune activation. Additionally, context-specific modulation, such as the use of TLR antagonists or neutralizing antibodies against inflammatory cytokines in autoimmune conditions, may further refine therapeutic outcomes. Personalized approaches, including identifying patient-specific immune signatures, can also help balance immunostimulatory effects with safety, reducing adverse reactions. Advancing these strategies is crucial to fully harness the therapeutic potential of NA-sensing TLRs across a broad spectrum of diseases.

In conclusion, the inflammatory nature of NA-sensing TLRs has driven the development of NA-sensing TLR based strategies to manage a wide range of immune-related diseases. However, the high degree of homology among different NA-sensing TLRs necessitate the careful design of agonists and antagonists to ensure selective targeting of pathogenic pathways without compromising overall immune function. Furthermore, the complexity of TLR signaling networks and their interactions with other immune components of the immune system underscores the need for a comprehensive understanding when developing novel therapeutic approaches to minimize unintended immunological consequences. Continued research in this area may pave the way for the development of more selective or broader-spectrum NA-sensing TLR modulators, ultimately expanding the range of therapeutic options for treating both infectious and non-infectious immune-mediated diseases.

## Data Availability

Not applicable.
